# Transient Ischemic Attack in the Setting of Carotid Atheromatous Disease with a Persistent Primitive Hypoglossal Artery Successfully Treated with Stenting: A Case Report

**DOI:** 10.7759/cureus.464

**Published:** 2016-01-21

**Authors:** Meng Huang, Marc Moisi, Michael E Zwillman, John J Volpi, Orlando Diaz, Richard Klucznik

**Affiliations:** 1 Department of Neurosurgery, Houston Methodist Neurological Institute; 2 Neurosurgery, Swedish Neuroscience Institute; 3 Anesthesiology and Critical Care, Houston Methodist Hospital; 4 Neurology, Houston Methodist Neurological Institute; 5 Radiology, Houston Methodist Neurological Institute

**Keywords:** persistent, hypoglossal, tia, atherosclerosis, stent

## Abstract

Fetal brain perfusion is supplied by the primitive dorsal aorta anteriorly, longitudinal neural arteries posteriorly, and anastomotic transverse segmentals. Most notable of these connections are the primitive trigeminal, otic, hypoglossal, and proatlantal arteries. With cranial-cervical circulatory maturation and development of the posterior communicating segments and vertebro-basilar system, these primitive segmental anastomoses normally regress. Anomalous neurovascular development can result in persistence of these anastomoses. Due to its territory of perfusion, the persistent primitive hypoglossal artery (PPHA) is associated with vertebral artery and posterior communicating artery hypoplasia or aplasia. As a consequence, primary blood supply to the hindbrain comes chiefly from this single artery. Although usually clinically silent, PPHA is susceptible to common cerebrovascular disorders including athero-ischemic disease and saccular aneurysmal dilation to name a few. We present a case of transient ischemic attack in a patient with a PPHA and proximal atherosclerotic disease treated by endovascular stenting.

## Introduction

The primitive hypoglossal artery (PHA) is a fetal developmental vessel that traverses the hypoglossal canal and is one of four main arteries that serves as a transverse segmental anastomosis between the primitive dorsal aorta and the longitudinal neural arteries prior to their maturation into the anterior carotid and posterior vertebrobasilar circulations. The PHA typically regresses by 40 days after gestation, but it can persist in very rare cases. The persistent primitive hypoglossal artery (PPHA) was previously thought to represent an asymptomatic radiographic finding; however, there is increasing literature highlighting the vascular pathologies associated with this anatomic variant [1-8].

Here we present a patient who was evaluated for transient neurological symptoms and found to have a PPHA originating just distal to the internal carotid take off with an associated greater than 60% stenosis and ulceration of the ICA segment proximal to it. The patient was treated by endovascular stent placement with embolic protection.

## Case presentation

**Figure 1:**
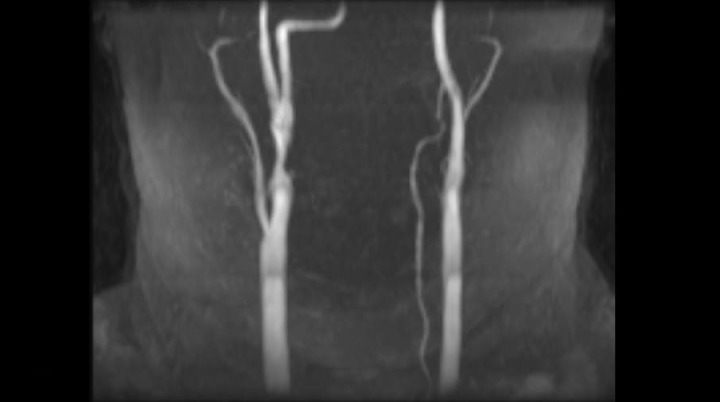
MRA of the neck showing the anomalous anastomosis of the right vertebral artery to the internal carotid artery segment 2.0 cm distal to the right common carotid artery bifurcation.

**Figure 2:**
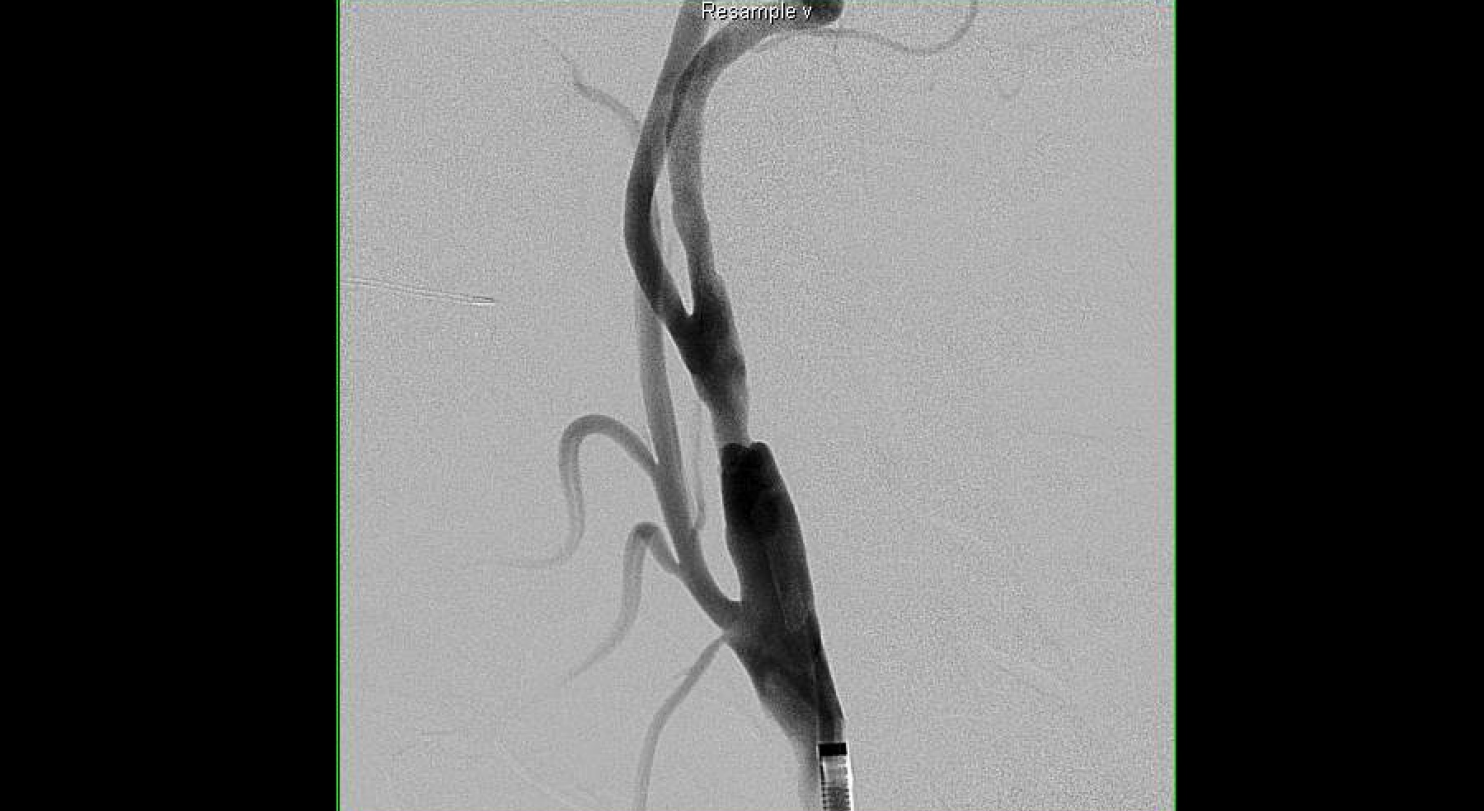
Oblique right common carotid artery injection before stent placement showing 1.3 cm length of plaque and greater than 60% diameter stenosis of the ICA segment proximal to the anomalous bifurcation.

**Figure 3:**
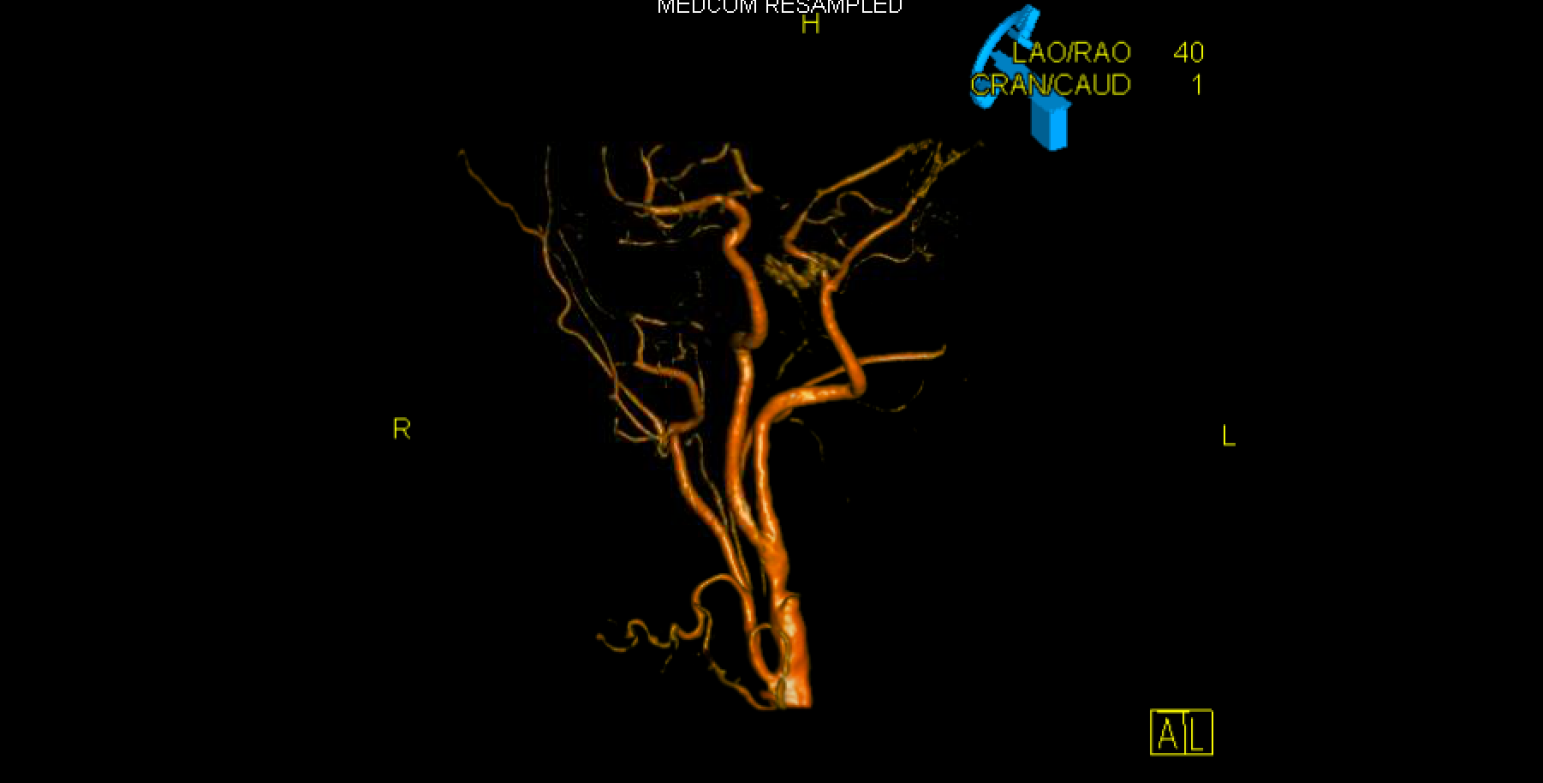
3D reconstruction identifying the anomalous vessel and proximal plaque.

**Figure 4:**
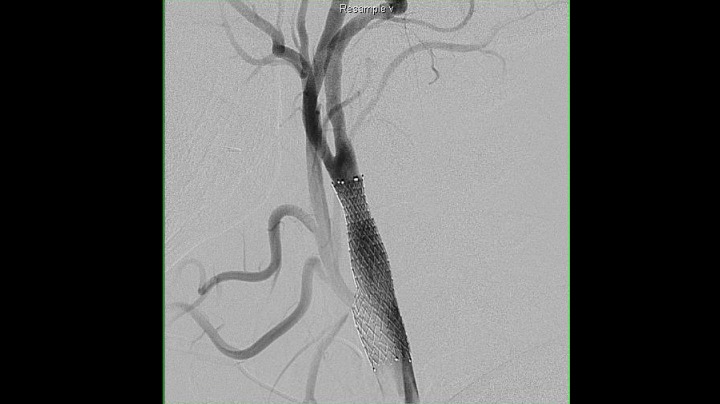
Oblique right common carotid artery injection after stent placement showing a restored normal luminal diameter and good flow across the ICA.

A 50-year-old female with a history of smoking and hypertension developed the acute onset of left upper extremity paresthesias and weakness lasting approximately 1 to 5 minutes with jaw numbness for another 5 minutes. All her symptoms resolved by the time she arrived at the hospital, and she was normotensive with no neurologic deficits on examination. A CT scan obtained in the emergency room showed a small chronic cortical insult of the ipsilateral temporal lobe, which was presumed to be unrelated. MRI of the brain showed no acute ischemia. MRA of the head and neck was then obtained and showed an anomalous anastomosis of the right vertebral artery to the internal carotid artery segment 2.0 cm distal to the right common carotid artery bifurcation (Figure 1). Formal digital subtraction angiography confirmed this anatomic variant originating from the right internal carotid and terminating as the basilar artery. Injection of the right, internal carotid thus demonstrated filling of the entire right hemisphere and posterior circulation (except the posterior inferior cerebellar artery (PICA)) from the right internal carotid. The absence of posterior communicating arterial flow was noted bilaterally. The vertebral arteries terminated at the PICA bilaterally. Just proximal to the ICA-PPHA bifurcation, there was a 1.3 cm length of plaque resulting in greater than 60% diameter stenosis (Figures 2-3). Given the clinical implications of atheroembolic disease affecting the only supply to the patient's brainstem and the technical difficulty of surgical endarterectomy with difficult anatomy, the patient was entered into the treatment arm of the SAMMPRIS trial and underwent endovascular stenting. After placement of a SpiderFx™ embolic protection device into the PPHA, a 7 x 10 x 40 mm Protege stent was deployed successfully across the stenotic segment and resulted in complete restoration of luminal diameter without the need for angioplasty (Figure 4). The patient tolerated the procedure well, was maintained on antithrombotics, and incurred no further ischemic symptoms in long-term follow up with the last angiogram one-year post-treatment showing stable resolution of stenosis and ulcerated plaque.

## Discussion

Persistent direct anastomoses between the carotid and basilar arterial systems are rare [9-10]. During early embryological development, carotid–basilar anastomoses serve to perfuse the posterior circulation while the vertebrobasilar system develops and matures. These vessels involute with the emergence of the posterior communicating arteries, usually by the 40^th^ day of fetal development [11-12]. Initially summarized by Lie [13], these communicating vessels include the trigeminal, otic, hypoglossal, and proatlantal arteries. The first three are named after the cranial nerves they accompany. The persistent trigeminal artery (PTA) is the most common of these anomalies, found in 0.1 – 0.2% of cerebral angiograms [14]. The persistent primitive hypoglossal artery (PPHA) is the second most common, found in 0.027 – 0.1% of cerebral angiograms [12,15].

The diagnostic criteria for a PPHA are as follows: 1. It arises from the ICA above the level of C3; 2. The PPHA enters the posterior fossa through the condyloid (hypoglossal) canal; 3. The ipsilateral posterior communicating artery is absent or hypoplastic; and 4. The basilar artery only fills distal to the junction with the PPHA [12-13,16]. Additionally, the vertebral artery is usually absent or hypoplastic either ipsilaterally or bilaterally [13]. It is rare to have all of these features together as did our patient [17].

MR angiography and conventional digital subtraction angiography are both crucial for defining aberrant anatomy in this case. A 3D reconstruction clearly identified the path of the anomalous vessel through the hypoglossal canal and helped differentiate it from its closest related variant, the persistent proatlantal intersegmental artery which enters the skull through the foramen magnum (Figure 3). Digital subtraction angiography provides critical, dynamic-flow-related information and allows identification of collateral circulation, if any, and the lack thereof as in our case. 

The PPHA is usually a benign, incidental finding on radiological imaging obtained for other clinical indications. When symptomatic, PPHAs have been associated with TIA, vertebrobasilar headache syndrome, a bowhunter-like dynamic syncopal syndrome, glossopharyngeal neuralgia, aneurysmal subarachnoid hemorrhage, and atheroembolic cerebrovascular accident [1-3,5,12,15,18-20]. Its identification is essential in the setting of symptomatic carotid atherosclerotic disease and must be taken into consideration when planning carotid procedures such as endarterectomy or stenting. 

Carotid endarterectomy in the management of ICA stenosis in the setting of PHA has been described by previous authors [7-8,21-23]. In the presence of a PPHA, this procedure incurs unique risks of high carotid exposure, identification of the PPHA during dissection, the challenge of cerebral perfusion maintenance with intraoperative shunting in the setting of posterior communicating artery and vertebral artery hypoplasia and or aplasia, and finally the arteriotomy closure given the proximity of the PPHA take off [12,22-23]. There are limited reports of cases of successful endovascular stenting [7-8,17,22-23].

In our case, the patient presented with symptoms of a transient ischemic attack attributable to ipsilateral carotid atheromatous disease proximal to a PPHA supplying the entire basilar and posterior cerebral circulation with aplastic posterior communicating segments and vertebral arteries terminating in PICA. Given the burden of atheromatous disease proxmial to the PPHA and risks of catastrophic brainstem infarction, endovascular lesional stenting was pursued in light of the surgical challenges. The patient was enrolled into the SAMMPRIS trial and underwent Protege™​ stent placement across the stenotic ulcerated segment with a concomitant SpiderFX™ embolic protection device deployed in the PPHA. The stent restored normal luminal diameter and flow successfully without complication or neurological sequela (Figures 2, 4). Angioplasty was not required or performed.

## Conclusions

PPHA with associated atherosclerotic disease is a difficult and potentially dangerous condition given the concomitant underdevelopment of carotid-basilar anastomoses and vertebral arterial systems. Surgical treatment, while feasible and successful in many reported cases, can pose significant risk. Limited reports show successful endovascular treatment with stent placement. Here we contribute to the growing body of literature highlighting successful endovascular (Protege™) stenting of a patient with symptomatic carotid atherosclerotic disease associated with a PPHA.
